# Preparation, characteristics and cytotoxicity of green synthesized selenium nanoparticles using *Paenibacillus motobuensis* LY5201 isolated from the local specialty food of longevity area

**DOI:** 10.1038/s41598-022-26396-4

**Published:** 2023-01-02

**Authors:** Qian Long, Lan-kun Cui, Sheng-bin He, Jian Sun, Quan-zhi Chen, Hao-dong Bao, Teng-yue Liang, Bao-yue Liang, Lan-yu Cui

**Affiliations:** 1 Key Laboratory of Longevity and Aging-Related Diseases of Chinese Ministry of Education, Guangxi Colleges and Universities, Key Laboratory of Biological Molecular Medicine Research, School of Basic Medical Sciences, Guangxi Medical University, Nanning, 530021 Guangxi People’s Republic of China; 2 Department of Clinical Laboratory, The Fourth People’s Hospital of Nanning, Guangxi AIDS Clinical Treatment Center (Nanning), No. 1 Erli, Changgang Road, Nanning, 530023 Guangxi People’s Republic of China; 3 School of History and Archive, Yunnan University, Kunming, 650000 Yunnan People’s Republic of China

**Keywords:** Biochemistry, Biological techniques, Biotechnology, Microbiology, Environmental sciences, Engineering, Materials science

## Abstract

Selenium is an essential micronutrient element. For the extremely biotoxic of selenite, Selenium nanoparticles (SeNPs) is gaining increasing interest. In this work, a selenium-enriched strain with highly selenite-resistant (up to 173 mmol/L) was isolated from the local specialty food of longevity area and identified as *Paenibacillus motobuensis* (*P. motobuensis*) LY5201. Most of the SeNPs were accumulated extracellular. SeNPs were around spherical with a diameter of approximately 100 nm. The X-ray photoelectron spectroscopy and Fourier transform infrared spectroscopy showed that the purified SeNPs consisted of selenium and proteins. Our results suggested that *P. motobuensis* LY5201could be a suitable and robust biocatalyst for SeNPs synthesis. In addition, the cytotoxicity effect and the anti-invasive activity of SeNPs on the HepG2 showed an inhibitory effect on HepG2, indicating that SeNPs could be used as a potential anticancer drug.

## Introduction

Selenium (Se) is an essential trace element, which forms at least 25 selenoproteins. These proteins involve in antioxidant, catalytic, anti-inflammatory, immunity and antitumor functions^1^. Se may promote longevity through diet^2^. Moreover, longevity area and selenium rich area showed significant positive correlation^3^. However, excessive intake of selenium can cause adverse effects^4^. Selenium nanoparticles (SeNPs) could overcome the high dosages of Se metal and keep the biological activities such as the anticancer^5^ and antibacterial properties^6^, making SeNPs particularly useful for pharmaceutical and biomedical applications.

Selenium nanoparticles (SeNPs) have gained attention in the electronics and optics industries for their special physical characteristics, such as photoelectric, X-ray sensing properties, and catalytic properties^7^. Physico-chemical methods for nanoparticle synthesis are costly, cumbersome, and generate hazardous by-products^8^, which hindered the wide application of SeNPs. Biological synthesis is considered to be the most ideal method for green synthesis. Microorganisms and plants were used to synthesize low-cost, energy efficient, environmentally friendly nanomaterials without toxic by-products^9^. This is a sustainable bottom-up synthesis method and it is easy to scale up. Green synthesis of nanoparticles is prefered to physical and chemical methods^10^. It has been reported that some bacteria, fungi and actinomycetes have the ability to produce nano-selenium from higher oxidation selenium, and in these populations, bacteria have been widely studied for their advatages such as rapid growth and strong operability^11^. Additionally, they could provide products with unique size and morphology^6^.A number of microbes could biosynthesize SeNPs intracellularly or extracellularly during the reduction of selenium oxyanions to elemental selenium^12,13^, providing a simple and environment-friendly method to prepare SeNPs. However, the selenite tolerance of most reported SeNPs producing microbes is relatively low (< 100 mmol/L)^14,15^, and the time for the reduction of these toxic forms is long, ranging from 24 to 96 h^16^. Thus, the identification of novel strains with a high selenium tolerance is urgently needed.

In this study, a new Se-reducing bacteria isolate (LY5201) showing tolerance to selenite (173 mmol/L, 30 g/L) was isolated from Chinese Sauerkraut, in Bama, “the hometown of longevity” in the world. We found that SeNPs could be synthesized by *Paenibacillus. motobuensis* (*P. motobuensis*) LY5201 efficiently under sodium selenite (Na_2_SeO_3_) stress in anaerobic conditions. SeNPs could be detected within 24 h, which is faster than reported microbes, indicating that this strain is suitable for SeNPs preparation. The SeNPs characteristics were determined by transmission electron microscopy (TEM) and fourier-transform infrared (FTIR) spectroscopy. Hereafter, the cytotoxicity of SeNPs was investigated. The SeNPs synthesized by *P. motobuensis* LY5201 may be used as a promising drug for anticancer.

## Materials and methods

### Selenite-reducing strain isolation and identification

Samples were isolated from Chinese Sauerkraut (Bama, Guangxi, China). The supernatant of the Chinese Sauerkraut was plated on Luria Bertani (LB) agar containing 500 mg/L sodium selenite. After incubated at 37 °C for 24 h, individual colonies are red, indicating Se reduction and Se^0^ formation. The single colony was confirmed by 16S rRNA gene sequencing analysis^17^. Isolate 201 (named LY5201) was selected for further study for its rapid growth rate and Se reduction performance. The 16S rRNA gene was amplified and sequenced as previously study^18^. The 16S rRNA gene sequence was compared to previously published sequences present on the EzBioCloud server^19^. A phylogenetic tree was constructed using the maximum likelihood algorithms of MEGA 7^20^.

### Assessment of sensitivity of LY5201 to Na_2_SeO_3_

The influence of SeNPs on the proliferation ability of LY5201 was investigated. First, a fresh overnight cell culture without Na_2_SeO_3_ was used as seed. Different concentrations of Na_2_SeO_3_ (0, 0.5 g/L, 1.0 g/L, 2.0 g/L, 5.0 g/L, 10.0 g/L and 30.0 g/L) were prepared in LB media. The seed was added into these LB media at an initial cell density of 0.3 (OD_600_). All cultures were incubated at 37 °C with rotary shaking at 200 rpm for 24 h. The experiment was done in triplicates for verifying the obtained results.

### Characterizations of SeNPs

In order to obtain SeNPs, the sterilized sodium selenite solution was added to the fermentation medium, bringing the final concentration of the solution to 5.0 g/L. The fermentation broth was centrifuged at 15,000 × g for 10 min. The resultant pellet was washed three times and resuspended in 20 mL deionized water. To separate the SeNPs from the cell fragments, 5 mL of 1-octanol was added. The mixture was mixed thoroughly and centrifuged at 2,000 × g for 5 min. Then, the mixture was placed at 4 °C for 24 h. The lower layer containing SeNPs was collected and cleaned consecutively with 70% ethanol, chloroform and deionized sterile water and freeze-dried^21^.

To further characterize the SeNPs, transmission electron microscopy (TEM, JEOL-7100), X-ray photoelectron spectroscopy (XPS, Escalab 250Xi), fourier transform infrared spectroscopy (FTIR, 640-IR), dynamic light scattering (DLS, Litesizer 500) and UV–visible analysis (UV–Vis, Varian Cary 100) were carried out as previously described^13^.

### Cytotoxicity analysis of SeNPs in vitro

Cell Counting Kit-8 (CCK-8, Beyotime Biotechnology, Shanghai, China) was used for in vitro cytotoxicity testing of SeNPs. Hepatocarcinoma (HepG2) purchased from the Cell Bank of Chinese academy of Sciences (Shanghai, China) was cultured in DMEM supplemented with 10% fetal bovine serum in 96-well plates. In this experiment, 96 well plates (200 μL media per well) were seeded with cells at final concentration of 1 × 10^5^ cells/mL and incubated in 5% CO_2_ incubator at 37 °C. After 24 h incubation, SeNPs were added into the culture to keep the final concentrations at 5, 10, 15 and 20 μg/mL The culture was wells incubated in 5% CO_2_ incubator at 37 °C for 24 h. Then, each well was treated with 10 μL of CCK-8 and incubated at the same previous condition for 2 h. The color intensity of the solution was measured at 450 nm using a multimode microplate reader (SuPerMax 3000FL, China). The percentage of cell viability was measured according to the following equation:

$${\text{Cell}}\;{\text{viability}} = \left( {{\text{A}}_{{\text{t}}} /{\text{A}}_{{\text{c}}} } \right) \times 100$$

where A_t_ represents the mean absorbance of cells treated with SeNPs, and A_c_ represents the mean absorbance of cells without SeNPs. Data are mean of triplicate experiments.

### Wound healing assay

The scratch wound assay was used to measure the effect of SeNPs on the migration ability of HepG2^21,22^. Cells were cultured in 6-well plates. After 24 h, the cell monolayer was scratched with a 10 μL pipette tip to create a gap. After washing three times with serum-free medium, the gap was photographed to determine the wound baseline. Cells were incubated in FBS-free media with SeNPs solution at different concentrations. The wound gap size were evaluated at 24 h post-wounding. Image J was used for wound healing assay. The healing ratee was calculated according to the formula as following^21^:

$${\text{Healing}}\;{\text{ratio (\% )}} = \left( {{\text{A}}_{0} - {\text{A}}_{{\text{f}}} } \right)/{\text{A}}_{0} \times 100\% ,$$

where A_0_ represents the area of initial wound area, A_f_ represents the remaining area of wound at 24 h.

### Statistical analyses

Statistical analyses were carried out by GraphPad Prism 9. One-way analysis of variance (ANOVA) followed was used to analyze the data. All data were presented as mean ± S.E.M. (n = 5). Significant differences were indicated with *P* value, *P < 0.05, **P < 0.001, ****P < 0.0001.

### Ethical approval

This is an observational study. The “Preparation, characteristics and cytotoxicity of green synthesized selenium nanoparticles using Paenibacillus motobuensis LY5201 isolated from the local specialty food of longevity area” Research Ethics Committee has confirmed that no ethical approval is required.”

## Results and discussion

### Isolation and Identification of LY5201

**Figure 1:**
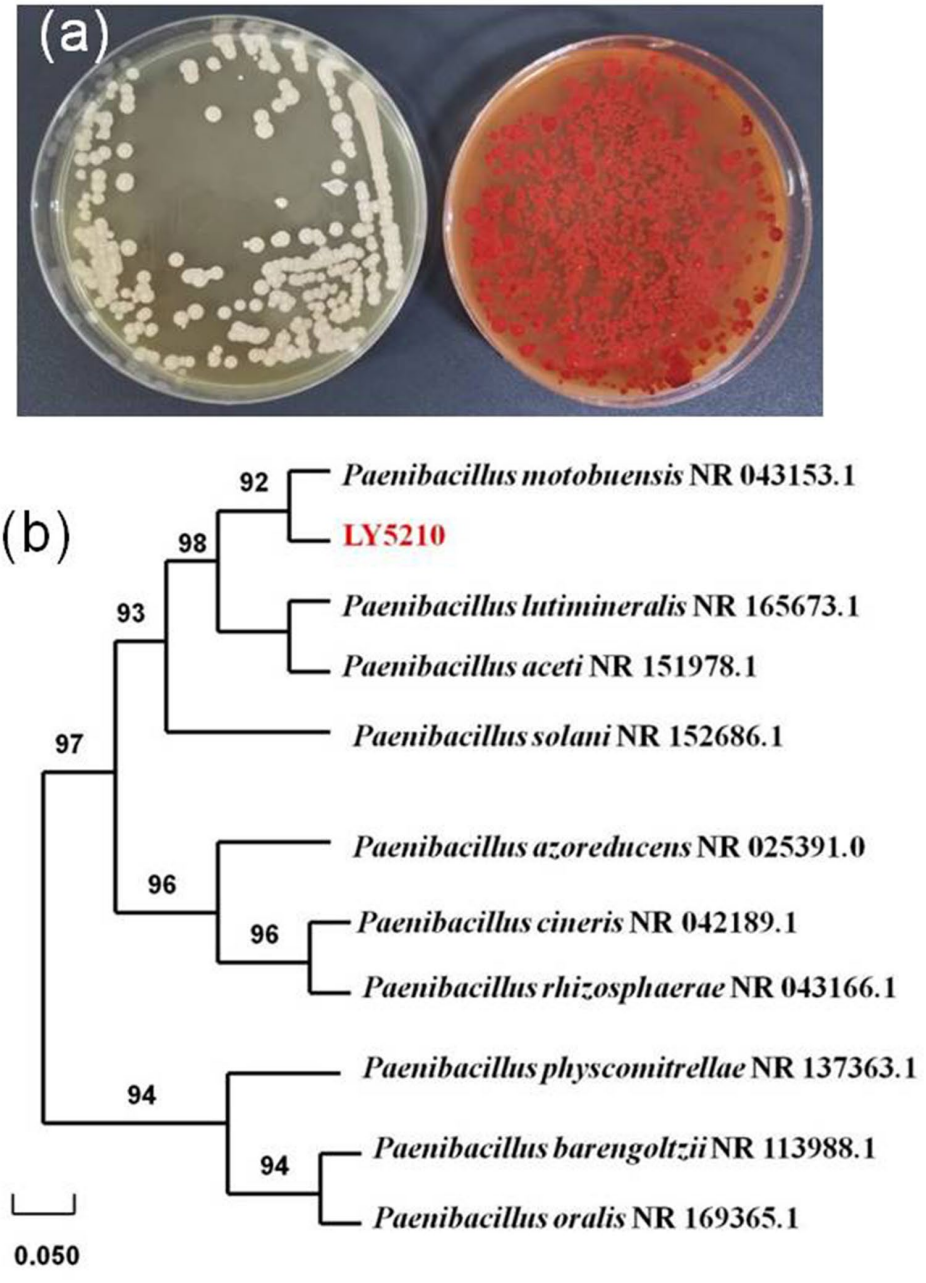
Growth of strain LY5201 on LB agar plates and phylogenetic analysis of the strain LY5201. (**a**) Images of cultures of strain LY5201 grown in absence (left) and presence (right) of 5 mmol/L Na_2_SeO_3_. The red colony color indicates selenite reduction and the formation of elemental selenium (Se^0^). (**b**) Maximum likelihood tree based on the 16S rRNA gene sequence of isolate LY5201. The scale bars indicate 0.05 substitutions per site.

Bama is “the hometown of longevity” in the world. Diet with adequate nutrition is one of the most important factors to longevity^23^. Trace elements play an important role in maintaining metabolic homeostasis in the elderly^24^. Since selenium rich area are related with longevity area, we tried to isolate the selenium-enriched strain from the local specialty food (Sauerkraut). In this study, LY5201 was isolated using LB plate supplemented with 5 mmol/L sodium selenite and exhibited the ability to reduce selenite to red Se^0^ (Fig. 1a).

As shown in Fig. 1b, 16S rRNA gene sequence and phylogenetic evolution analysis showed that LY5201 have a high degree of similarity (92%) with *Paenibacillus motobuensis* NR 043,153.1. It also exhibited the typical biochemical characteristics of *P. motobuensis*^25^ as indicated in Table S1 (supporting data). The strain LY5201 was identified as *P. motobuensis* LY5210. It is Gram-negative, rod-shaped bacterium. In addition, this is the first study to prove that a *P. motobuensis* strain could reduce selenite to Se^0^ and biosynthesize SeNPs. Recent studies have reported that hydroxyapatite nanoparticles could be synthesized by *Acacia falcata* leaf extract^26^ and the fruit extract of *Spondias pinnata* could synthesize hematite nanoparticles^27^. It is considered that three steps were involved in the synthesis of nanoparticles by plants extracts: reduction of metal ions, nucleation and growth^28^. The secondary metabolites and biomolecules in plant extracts, such as carbohydrates and protein could reduce metal ions into stable nanoparticles and enhance their morphology. What’s more, the reduction potential of the polyphenolic compounds in the extracts of plant are sufficient to reduce metal oxide to zero valence, which yields nanoparticles. Unlike the cell-free system in plant extracts systerm, synthesis of nanoparticles from microbes usually based on the whole cell. The mechanism of SeNPs synthesized from Se^0^ varies among diverse microbial species including several metabolic pathways, enzymes and different proteins for the reduction process. It is considered that there are mainly three steps of SeNPs: (1) Transfer of selenite/selenate into the cells. (2) Reduction of elemental Se inside the cell to Se^0^.(3) Assembly of Se^0^ to SeNPs^29^.

### The selenite tolerance of LY5201

**Figure 2:**
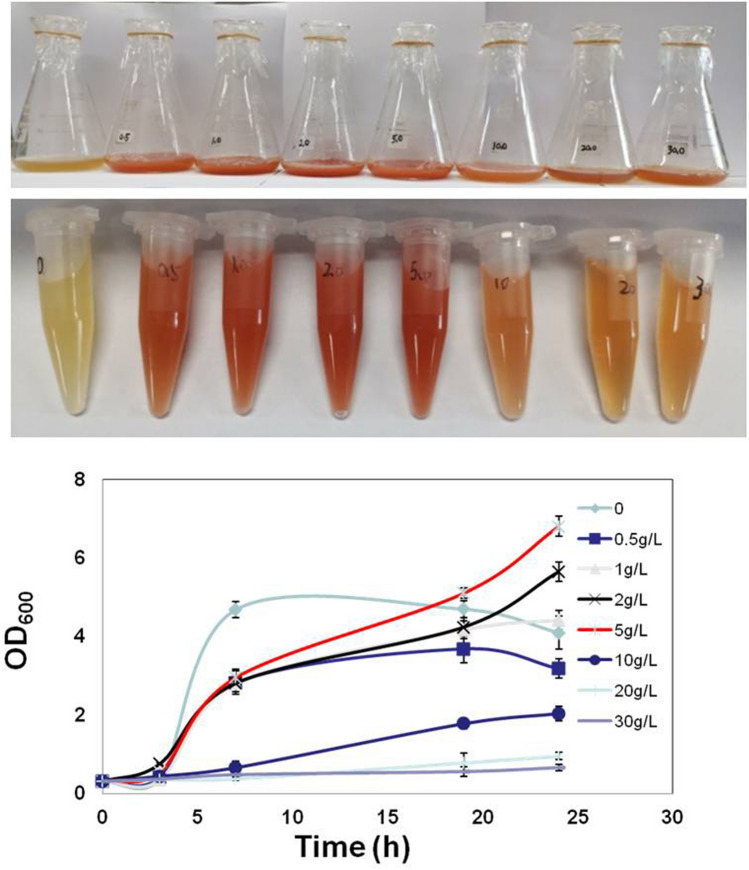
Growth of *P. motobuensis* LY5201 in different concentrations of Na_2_SeO_3_.

As shown in Fig. 2, to determine the selenite tolerance of LY5201, bacterial cells were grown in LB media with different concentrations of selenite, and the specific growth rates of cells were 2.81 ± 0.13 h^−1^, 1.46 ± 0.11 h^−1^, 1.74 ± 0.07 h^−1^, 1.67 ± 0.15 h^−1^, 1.09 ± 0.02 h^−1^, 0.19 ± 0.06 h^−1^, 0.11 ± 0.03 h^−1^ and 0.07 ± 0.02 h^−1^ in selenite concentrations of 0, 0.5 g/L, 1.0 g/L, 2.0 g/L, 5.0 g/L, 10.0 g/L, 20 g/L and 30 g/L, respectively. LY5201 coule tolerate selenite, it showed slightly growth in the presence of 30 g/L selenite concentration (173 mmol/L). Since elemental selenium scatters and absorbs radiation at 600 nm, which contributed to the increased absorbance^30^, the absorbance of the cell culture with selenite exceeded the value of control (0 g/L) and the growth rates at 1 g/L and 2 g/L selenite concentrations is higher than at 0.5 g/L selenite concentration. LY5201 has a higher level of selenite resistance than some other bacteria such as *Azoarcus sp*. CIB (8 mmol/L)^31^ and *Rhodopseudomonas palustris* (8 mmol/L)^32^, which is similar to the selenite-tolerant strain, such as *Alcaligenes faecalis* (20.7 g/L)^13^.

### Characterizations of P. motobuensis LY5201 and SeNPs

**Figure 3:**
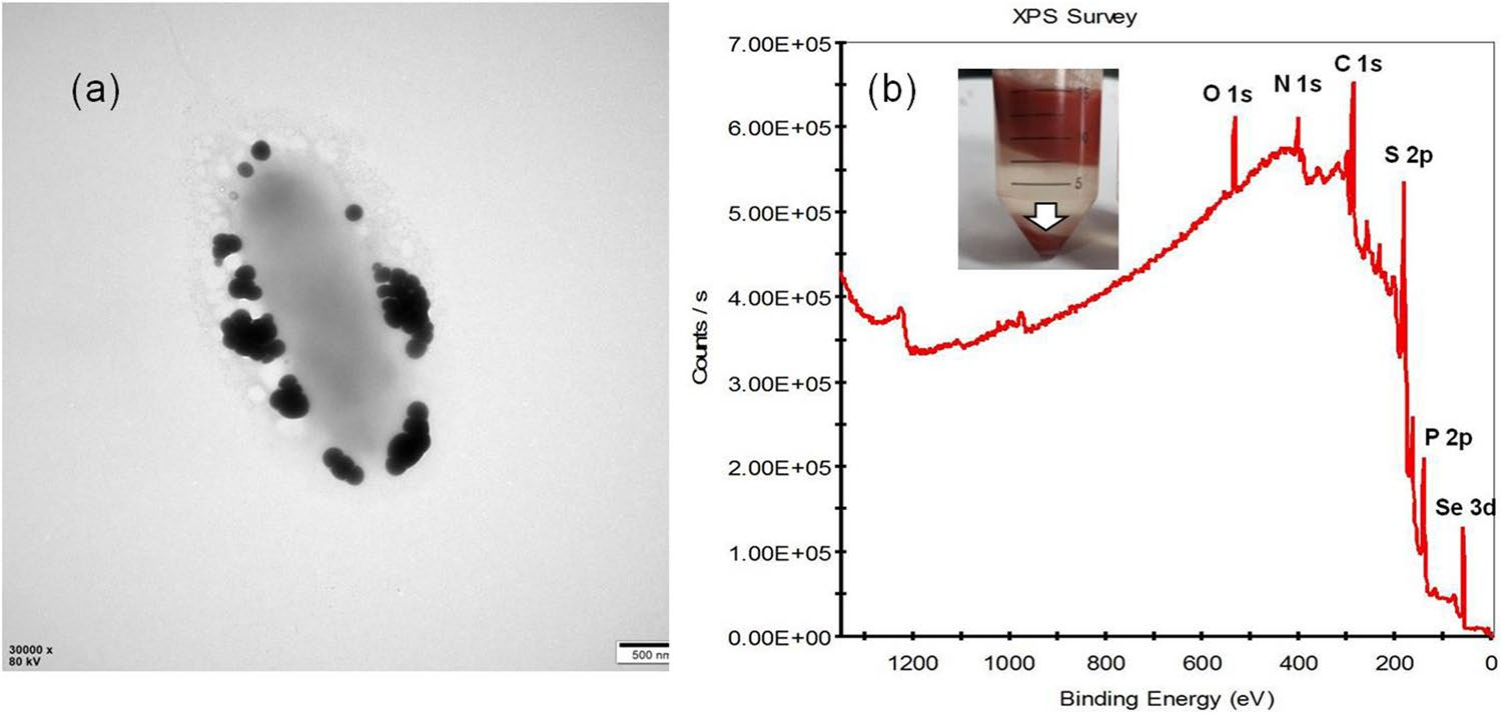
Characterizations of *P. motobuensis* LY5201 and SeNPs (**a**) TEM micrographs showing SeNPs produced by *P. motobuensis* LY5201 at 24 h. (**b**) Extracted biomolecules capped-SeNPs contained C, N, O, Se, S and P elements by X-ray photoelectron Spectroscopy (XPS) analysis.

The purified SeNPs were characterized. TEM was used to determine the location of SeNPs produced by *P. motobuensis* LY5201(Fig. 3a). The naoparticles were located in the extracellular spaces. The particles were spherical and had homogenous size distribution. The average size of SeNPs was about 100 nm, similar to those found in *Providencia rettgeri*^16^ and *Lactobacillus casei*^21^. More researches are needed to elucidate whether vesicular secretion is involved in the formation of SeNPs.

**Figure 4:**
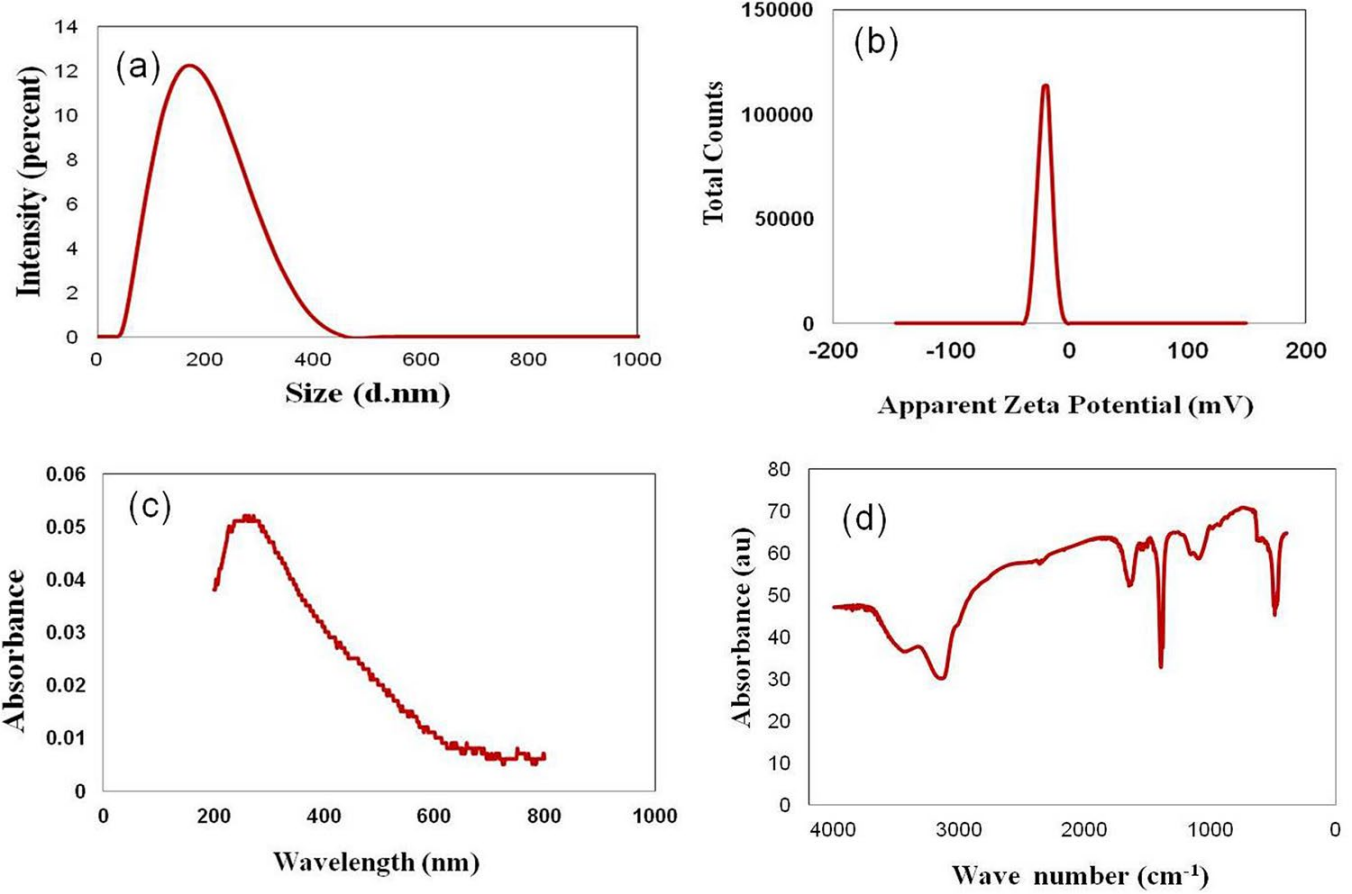
Characterizations of SeNP_S_. (**a**) Size distribution of SeNPs. (**b**) zeta potentials of SeNPs. (**c**) UV–visible spectra of SeNPs in distilled water. (**d**) FTIR spectra of SeNPs.

The XPS spectra showed that signals of C, O, N, S, P and Se were detected (Fig. 3b). The average particle size of SeNPs was obtained from the DLS analysis (Fig. 4a). The average poly dispersity index (PDI) was 0.227. Lower the PDI, the lesser the aggregation of particles. The main average size of the SeNPs was found at 130.4 ± 12.34 nm, which is larger than that in TEM analysis. It is possible that the size obtained from DLS does not only depend on the metallic core of SeNPs but also affected by the substances located on the surfaces such as bio-moieties and proteins^4^. The average size of SeNPs indicating that it can be used for biomedical applications such as biomaterial and bioactive drug deliver. As shown in Fig. 4b, SeNPs showed a negative zeta potential (-21.6 mv), indicating their stability in water. The presence of negatively charged functional groups on the surface of SeNPs were responsible for negative values of zeta potentials^33,34^.

The absorption spectra of SeNPs was shown in Fig. 4c. SeNPs exhibit a broad absorption peak at approximately 260 nm corresponds to previous study^7^, which confirms the formation of SeNPs. The UV spectra centered between 200 and 300 nm was due to the formation and surface plasmon vibration of SeNPs^35^. As shown in Fig. 4d, the FTIR spectra of the SeNPs showed absorption peaks at 3218 cm^−1^, 1571 cm^−1^, 1385 cm^−1^, 1313 cm^−1^, 1063 cm^−1^, 770 cm^−1^ and 559 cm^−1^. The peak at 3218 cm^−1^ corresponds to the O–H stretching, or to the N–H asymmetric stretch of proteins^4,33^. The peak at 1571 cm^−1^ corresponds to the amide II band of proteins^36^. Band at 1385 cm^−1^, 1313 cm^−1^ and 1063 cm^−1^ are assigned to C–N bond of aromatic and aliphatic amines^4^. Peaks at 770 cm^−1^ and 559 cm^−1^ may refer to the binding of SeNPs with –OH as Se-O, which indicating the SeNPs is modified by other chemicals.

### The effects of SeNPs on HepG2

**Figure 5:**
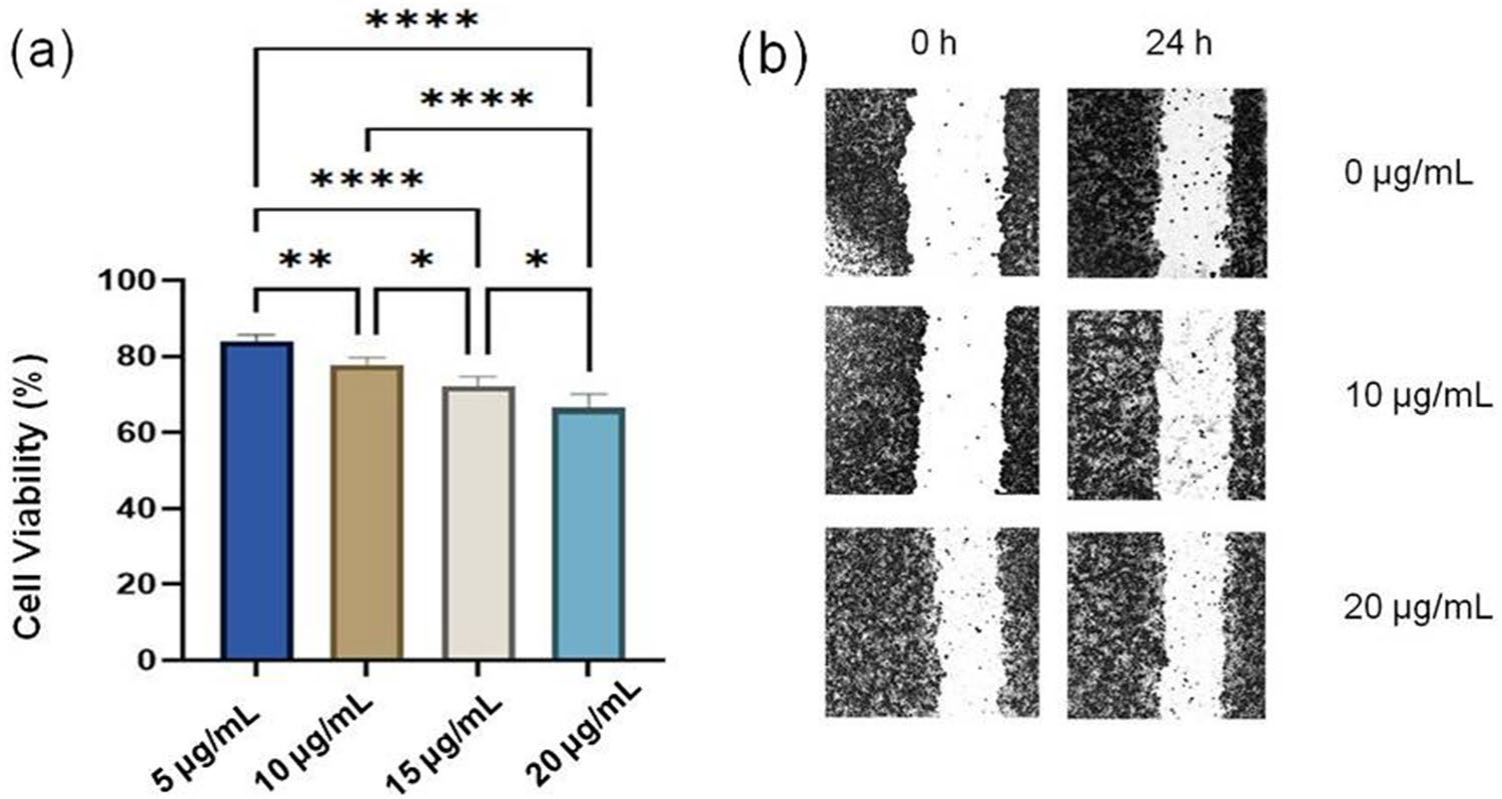
The effects of SeNPs on HepG2. (**a**) The cytotoxicity of SeNPs on HepG2 was determined by CCK-8 assay (*P < 0.05, **P < 0.001, ****P < 0.0001). (**b**) Cell migration was examined by wound healing assays.

The cytotoxicity of SeNPs was evaluated in vitro against the HepG2 cell line at four doses: 0, 5, 10, 15 and 20 μg/mL. The viability of HepG2 cells were ranging from 83.9 ± 1.58% to 66.4% ± 6.94%. There are significant differences in cell viability among these groups (*p* < 0.05) (Fig. 5a).

The scratch wound assay was carried out to evaluate the effect of SeNPs on the migration of HepG2 (Fig. 5b). Cell migration was examined via wound healing assay. SeNPs at different concentrations showed inhibitory effects on the migration of HepG2. The percentages of open wound were 31.52%, 21.71% and 1.86% in cells treated with SeNPs at 0, 10 and 20 μg/mL in a dose-dependent manner. SeNPs had been reported to have an anticancer activity on kidney, lung, liver and breast^12^. The anticancer mechanism of nanoparticles involves overproduction of ROS, depletion of mitochondrial membrane potential, and intracellular enzyme and protein interactions^37^. Oxidative stress can induce the cytotoxicity of nanoparticles to cancer cells, and DNA damage and cell mutation can lead to the damage of cancer cell development^38^. The results of our study are consistent with the published reports. However, the mechanisms of anticancer are not fully understood, which depending on the synthesis method, the functional groups on the surface, size and the structure of SeNPs ^5,18,21,39^. The antitumor activity of SeNPs synthesized by *P. motobuensis* LY5201 should be further investigated in the future.

## Conclusion

The selection of the best biocatalysts to synthesize SeNPs in a fast and efficient manner is the most important factor in the SeNPs application. *P. motobuensis* LY5201 have the ability to synthesize extracellular SeNPs when growing with sodium selenite within 24 h. Organic-aqueous extraction is a successful method for collection of SeNPs. By this method, biomolecules modified-SeNPs was obtained. The SeNPs had cytotoxicity to HepG2 and inhibited the migration of HepG2. These results indicating that the SeNPs synthesized by *P. motobuensis* LY5201 may be used as a promising drug or biomaterial for hepatocellular carcinoma. *P. motobuensis* LY5201 could be used as a selenite bioconversion platform suitable for biological applications.

## Supplementary Information

Supplementary Information.

## Data Availability

The datasets generated during and/or analyzed during the current study are not publicly available as the data also forms part of an ongoing study, but are available from the corresponding author on reasonable request.
