# Supplementary material for: Preparation, characteristics and cytotoxicity of green synthesized selenium nanoparticles using Paenibacillus motobuensis LY5201 isolated from the local specialty food of longevity area
Source: Sci Rep. 2023 Jan 2;13:53. doi: 10.1038/s41598-022-26396-4 (PMC9807572; doi:10.1038/s41598-022-26396-4)
Supplement: Supplementary file 1 — Supplementary Information. [file 41598_2022_26396_MOESM1_ESM.doc]

Table S1. Characteristics of LY5201

| **Characteristic** | **LY5201** |
| --- | --- |
| Gram-staining | - |
| Oxidase | + |
| Growth at 50°C | + |
| Optimum growth temperature | 37°C |
| Utilization of N-acetylglucosamine | - |
| Utilization of L-Fucose | - |
| Utilization of Glycerol | + |
| Utilization of Sorbitol | - |
| Utilization of Mannitol | - |
| Utilization of Glycogen | - |
| Growth in NaCl at 5% | + |

Note: +, Positive; -, negative.


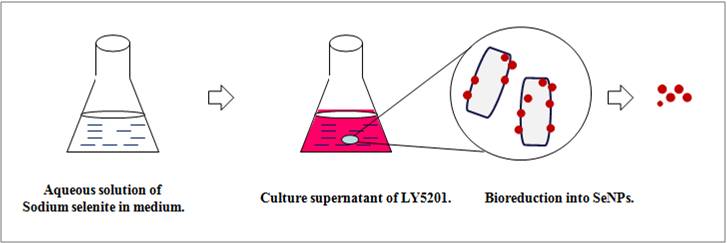


Fig. S1 Schematic diagram of selenium nanoparticles synthesis.
